# PROTOCOL: Language interventions for improving the L1 and L2 development of dual language learners in early education and care: A systematic review and meta‐analysis

**DOI:** 10.1002/cl2.1131

**Published:** 2021-07-10

**Authors:** Franziska Egert, Steffi Sachse, Katarina Groth

**Affiliations:** ^1^ State Institute of Early Childhood Research (IFP Bayern) Muenchen Germany; ^2^ Institute of Psychology University of Education Heidelberg Heidelberg Germany; ^3^ German Youth Institute (DJI) Muenchen Germany

## Abstract

The aim of the current systematic review and meta‐analysis is to comprehensively synthesize the effectiveness of language promotion interventions in early childhood education and care (ECEC) settings on L1 and L2 development of dual language learners (DLLs). We will use the PICOC‐Strategy (Population, Intervention, Comparison, Outcome, Context) suggested by Petticrew et al. to frame our research questions. Specifically, this review has the following research questions: (1) To which extent do language interventions accomplished in ECEC settings effect the L2 development (in the society language) of DLLs? (2) To which extent do bilingual language interventions (with L1 as language of instruction) accomplished in ECEC effect the L1 development (in the family language) of DLLs? (3) Are there significant differences in the effectiveness of different language interventions (additive vs. integrated vs. bilingual/two‐way‐immersion) to promote the L2 development of DLLs? (4) Are language interventions accomplished in ECEC settings more effective in supporting the L2 development (in the society language) of DLLs, when they start early in life (before the age of three)? (5) Are language interventions more effective in supporting the L2 development (in the society language) of DLLs when they are implemented with high fidelity/high quality? (6) Are language interventions with teachers as implementers more effective in supporting the L2 development (in the society language) of DLLs than language interventions with external implementers? (7) Are language interventions with higher intervention dosage (exposure × attention) more effective in supporting the L2 development (in the society language) of DLLs?

## 1 BACKGROUND

A large and growing number of students in Europe or the United States come from families where the societal language (L2) spoken in schools and day care centers is not the primary language (L1) spoken at home (Passel, 2011). These children grow up in environments with different languages. Dual language learners (DLLs) are a very diverse group of children: children who learn two or more languages more or less simultaneously and children who learn two or more languages successively, but in very different age ranges (i.e., who have made progress in learning one language when they begin the acquisition of another language). In many cases and especially relevant for this review are children who are faced with a different language at home than in society. This review is focused on DLLs where the language spoken at home (L1) differs from the language spoken in the society in which a child lives (L2).

### 1.1 Description of the condition

Growing up with two languages may cause variation in language development, but is not necessarily associated with developmental problems (Bialystok, 2001; De Houwer, 2009; Tracy, 2007). To acquire one, two or more languages, the exposure to all these languages is needed. In particular, quantitatively and qualitatively high language input has to be provided. However, the input children receive in their first (minority/family, L1) and second (societal, L2) language varies considerably. Therefore, the oral language skills in the society spoken language (L2) and also the minority/family spoken language (L1) vary significantly. Most DLLs enter school with L2‐skills and experiences that differ substantially from monolingual native speakers (Ballantyne et al., 2008). They often lag behind their monolingual native speaking peers in L2‐competencies due to varying input and very often also due to associated socio‐economic situations (Dubowy et al., 2008; Reardon & Galindo, 2006). In general, language‐minority students who cannot communicate proficiently in the society spoken language (mostly L2) cannot fully participate in schools, workplaces, and/or society (August & Shanahan, 2006). The oral language skills in the society spoken language of preschool years are very crucial to educational success. Competencies in the societal language provide the foundation for the later development of reading and writing (Weinert, 2008). A high proportion of DLLs lags behind monolingual native speakers in academic progresses (Stanat et al., 2002; Tienda & Haskins, 2011). Early childhood is seen as a critical period for children who are DLLs, because children are challenged in learning a new language while acquiring essential skills for school (Buysse et al., 2014).

Besides focusing on improving societal language (L2) skills, there should also be a focus on preserving the family (L1) language(s) of the children within the contexts of early childhood education and care (ECEC). Growing up with different languages and being bi‐/multilingual can be a great resource for cognitive and academic development (Barac & Bialystok, 2012; Engel de Abreu et al., 2012).

### 1.2 Description of the intervention

#### 1.2.1 Promoting learning in the society spoken language (L2) in ECEC

There are different strategies and theoretical approaches to promote language development in children within ECEC context. Further, several evaluations on the impact of language interventions for children at risk/children with low language abilities, especially in the society spoken language (L2), exist:

(1) “Integrated/embedded interventions” focus on the quality of teacher–child interactions and language modeling strategies that are provided by caregivers/teachers within daily routines of child care centers. Some results show beneficial effects of integrated interventions on language development of young children at risk (cf. Buschmann et al., 2010; Girolametto et al., 2003) and on DLLs (e.g., Schuler et al., 2015).

(2a) Very specific “additive curriculum‐based interventions” focus on one or two particular language structures and use standardized material and manuals (Groth et al., 2017; Sachse et al., 2012).

(2b) “Additive small group interventions” often address a specific group of children (e.g., DLLs) as well as different linguistic abilities like vocabulary or grammatical knowledge within more or less structured sessions. These sessions take place on one or several days per week (Auleear Owadally, 2015). Findings on the impact of additive language promotion programs with or without curriculum and standardized material are quite heterogeneous (e.g., Fricke et al., 2013; Hofmann et al., 2008; PCERI, 2008). Potential explanations of the null effects of additive language programs are implementation fidelity (e.g., in‐service teacher training, experience of teachers) and intervention fidelity (e.g., exposure, attention rates, and implementer). Many of those studies include DLLs in their samples, but only a few explicitly report findings for this specific group of children.

(3) Shared book reading and dialogic reading programs are a powerful tool to promote language learning for all children in ECEC (e.g., What Works Clearinghouse, 2007, 2010, 2015). Based on single studies results on DLLs, a small body of evidence supports the benefit of book reading interventions for second language acquisition (e.g., Chlapana & Tafa, 2014; Ennemoser et al., 2013).

(4) Bilingual or translingual programs are immersive and provide child care lessons and activities in both the society (L2) and minority (L1) spoken language to ensure the similar input. However, the bilingual instruction can be offered in part‐time segments or via code‐switching (Barik & Swain, 1975; Duran et al., 2015).

(5) Combination of the different approaches (e.g., dialogic reading, structured small group interventions) exist and pilot studies provide at least some preliminary positive finding on the L2 development of DLLs on pre‐ and posttest change scores (Buysse et al., 2011; Han et al., 2014). For example, DLLs in Head Start centers received a language curriculum (Doors to Discovery), daily book reading session to teach two vocabulary words a day, modified literacy center activities, and daily theme related small group interventions in the researcher modified curriculum (Han et al., 2014). The Recognition and Response approach offers small group activities, embedded strategies in daily routines and individual interventions for DLLs to support language learning (Buysse et al., 2011; LaForett et al., 2013).

#### 1.2.2 Promoting learning in minority/family language (L1) in ECEC

In order to promote not only the societal language (L2), but also the minority/family spoken languages (L1) of the children different approaches are suggested and implemented:

(1) Integrated/translingual interventions are implemented, which means that language input in the minority/family spoken language (L1) for a special language group of children is given by native speakers within the daily routine of the child care center (e.g., Spanish in the United States).
(2) Additive programs/interventions (e.g., book reading sessions) are performed in the minority/family spoken languages (L1) within special time slots during the week.

A complex and interacting relationship between the language systems of the L1 and L2 of children is suggested in literature (e.g., Brisk & Harrington, 2007; Cummins, 1979). However, studies evaluating pilot programs which attempt to promote the development in the L1 have produced inconsistent results regarding possible effect on L2 development. For example, no impact was found by Moser et al.  (2010) while small transfer effects were found by Leseman et al. ( 2009). A high proficiency level in both languages is considered as desirable (Chilla et al., 2010).

Considering the school readiness dilemma, there is a pressing practical need for more information about effective language promotion strategies and instructional practices for DLLs (Buysse et al., 2014; Lisker, 2010; Redder et al., 2011) in ECEC context. This is essential for researchers and practitioners in order to develop better interventions in early education settings to promote second language (L2) development. The planned systematic review and the following meta‐analysis can provide important insights on what works to foster language development of DLLs.

### 1.3 How the intervention might work

#### 1.3.1 Language acquisition of DLLs

Recent research points out that language development and second language acquisition is dependent on a great variety of factors, which can be found at multiple levels. There is substantial body of evidence that linguistic performance is influenced by child characteristics (e.g., age, gender, phonological memory skills; Flöter et al., 2013; Melhuish, 2010; Parra et al., 2011; Sammons et al., 2002; Verhagen & Leseman, 2016), family background (e.g., parental education, socioeconomic status (SES), language stimulating activities, family language (L1); Dubowy et al., 2008, 2009; Halle et al., 2012; Hammer et al., 2014; Hart & Risley, 1995; Hoff, 2013; Leseman et al., 2009; Prevoo et al., 2014) as well as the ecological environment with special importance of early education and care (see section below).

We will investigate several factors in the meta‐analysis, which are likely to moderate the effects of language promotion in ECEC. In particular, we will inspect the following:

- Starting age for intervention beginning
- Fidelity of intervention (quality and attention)
- Exposure (dosage/duration)
- Implementer (external implementer vs. teacher)

The *starting age for intervention beginning* could be a very important factor. Different types of intervention could be more or less appropriate for different age groups (Egert & Hopf, 2016) and an early start of language promotion in periods that are very sensitive for language learning in general and especially for DLLs could be more effective.

*Fidelity of intervention (quality and attention)* might play an important role in the estimation of language intervention effects. *Treatment fidelity*—the quality of program and curriculum implementation—is widely considered as a predictor for effects on child outcomes. Curriculum fidelity is the extent to which a curriculum is implemented as intended (Dane & Schneider, 1998). In general, higher fidelity scores are suggested to be related to better child outcomes (Domitrovich et al., 2010; Hamre et al., 2010). Low implementation fidelity of the targeted language promotion approaches (implemented by external staff or teachers) could lead to reduced effects as well as reduced “time on task” spent by the involved children (e.g., Sachse et al., 2012).

*Exposure in terms of dosage/duration* is likely to influence the direct outcomes of language interventions for DLLs. It is well documented, that time of exposure to the different languages of a DLL has an impact on the language abilities in general (e.g., Hoff et al., 2012) as well as within language promoting settings in ECEC (e.g., Groth et al., 2017).

Different types of language interventions can be carried out by *different implementers (external trainer vs. day care teacher)*. Language interventions in ECEC settings can be carried out by external implementers like scientific staff or external trained persons versus teachers of the facilities. Motivation, educational background, language specific knowledge etc. could differ between these two groups of implementers and lead to differential effects (Egert & Hopf, 2016).

#### 1.3.2 Logic model for predictors of language learning and language promoting interventions in ECEC

**Figure 1:**
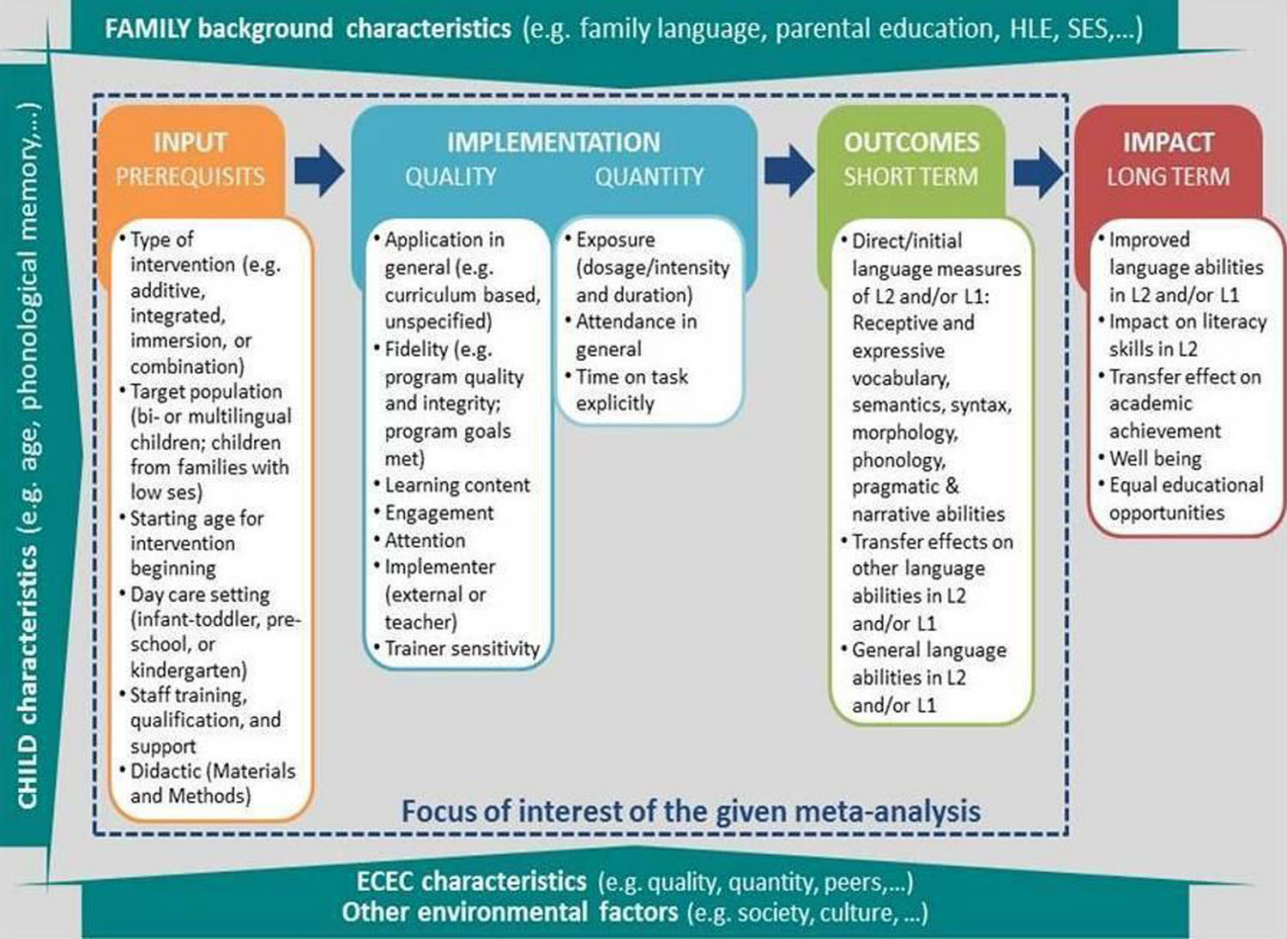
Logic model for L1/L2 language learning and promotion. ECEC, early childhood education and care; HLE, home learning environment; SES, socioeconomic status

The logic model in Figure 1 summarizes important factors which should be taken into account at the different stages of evaluation studies on L1/L2 intervention programs. It further summarizes the levels and factors, which might influence intervention effects and thus have to be considered as possible moderators in the given meta‐analysis. There are several factors, which are of special interest for the given meta‐analysis. In particular, variations in the effect size will be analyzed with regards to (1) input, (2) implementation of intervention, including the implementation process and its' quality (e.g., fidelity) as well as quantitative aspects (e.g., exposure), and (3) context factors like child and family characteristics as well as the ECEC context, as these factors influence language development in general. Additionally (4) methodological issues regarding the study design (like sample randomization, attrition rate (drop out), external or internal evaluation, reliability of outcome measures, effect size data to calculate effect sizes) have to be taken into account when estimating the effect size. However, as the methodological moderators refer to the study design and not to the intervention evaluation design, they are not depicted in this logic model.

### 1.4 Why it is important to do this review

An important ecological environment, in which language learning occurs, is center based care. According to OECD statistics, on average 86% of children between 3 and 5 years old are enrolled in ECEC and approximately 30% of children below 3 years attend child care in OECD countries around the world (OECD, 2018). A significant proportion of these children grow up with a family spoken language (L1) that differs from the society spoken language (L2). Demographic data as well as research shows that many of these children learn the societal language (L2) after entering day care (Sachse et al., 2010). Furthermore, about 20%–30% of the children entering school lag behind in their L2 abilities (Autorengruppe Bildungsberichterstattung, 2016; Ballantyne et al., 2008). This highlights the need for early and effective language interventions, since language abilities are extremely important for the further development of these children, especially for educational success (Stanat et al., 2002; Tienda & Haskins, 2011; Weinert, 2008). Besides learning the societal language (L2) there should also be a focus on preserving and developing the family soken (L1) languages of the children. Being successful bi‐/multilingual language learners can be a great resource for the development of these children. ECEC play a crucial role for the language development of DLLs. Up to now we do not know enough about effective strategies within early childhood education in order to promote first and second language abilities. So far, no specific systematic review has been completed on this topic.

Thus, specific features of early child care play a crucial role and should be taken into account as potential predictors of first and second language acquisition, respectively, development. Just as on the family level, high‐quality and ‐quantity, nonfamily care with good linguistic input significantly improves the language skills of children (for an overview see Sylva et al., 2011). A positive impact of early child care on language proficiency could be shown especially for migrant children and children from low SES‐families who only get limited L1 or L2 input at home (Beckh et al., 2014; Halle et al., 2012). Espinosa (2007) underlines the role of ECEC for DLLs since attendance offers more qualitative societal language (L2) experiences compared to received input at home. However, this positive impact on second language (L2) development was only observed for high‐quality early child care and was especially striking for migrant children from low SES‐families (Beckh et al., 2014). Thus, Stolarova et al. (2014) showed that lexical skills of 2‐year old children that are being cared for at home or in high‐quality day care centers do not differ if their parents have mid to high SES.

Finally, specific language promotion to foster the child's L1 or L2 abilities has to be considered as a further predicting factor. Nowadays, numerous language promotion programs on the level of day care settings are being applied to support the children's L1 or L2 development (for programs being provided in Germany see Lisker, 2011). However, implementation of these language intervention programs differs substantially and evaluations on the impact of such programs are either very heterogeneous (e.g., Egert, 2017; Egert & Hopf, 2016) or still lacking. Multiple explanations why intervention effects are not found or lacking are under debate (Ennemoser et al., 2013). In order to support children's language abilities, it is essential to specifically identify the factors which are important for an optimal and effective L1 and L2 improvement at an early age.

## 2 OBJECTIVES

The aim of the current systematic review and meta‐analysis is to comprehensively synthesize the effectiveness of language promotion interventions in ECEC settings on L1 and L2 development of DLLs.

We will use the PICOC‐Strategy (Population, Intervention, Comparison, Outcome, Context) suggested by Petticrew and Roberts (2005) to frame our research questions (Table 1).

**Table 1:** PICOC‐strategy

Population	DLLs from 0 to 6 years enrolled in ECEC. DLLs are all children living in a household where one or more members speak a language other than the society spoken language (L2). Exclusion of children with diagnosed language and speech disorders.
Intervention	All kind of specific language interventions applied in ECEC settings. Interventions have to be especially designed and targeted for DLLs to promote the society spoken language (L2). The minority/family language (L1) of the children spoken at home can also be promoted by these interventions, but this is not a requirement. Language interventions have to be implemented by teachers or external professionals. Exclusion of therapeutic interventions.
Comparison	DLLs from 0 to 6 years enrolled in ECEC receiving regular input in the society spoken language (L2) (business as usual). The comparison could also be a mandatory comprehensive language promoting program, in which all DLLs are enrolled in (alternative treatment).
Outcome	L1 development (in minority/family language) and L2 development (in the societal language)
Context	All kind of ECEC settings in centers and elementary schools including infant/toddler, preschool, pre‐K, kindergarten and mixed‐aged classrooms. Exclusion of family and home care settings.

Specifically, this review has the following research questions:

1. To which extent do language interventions accomplished in ECEC settings effect the L2 development (in the society spoken language) of DLLs?
2. To which extent do bilingual language interventions (with L1 as language of instruction) accomplished in ECEC effect the L1 development (in the family spoken language) of DLLs?
3. Are there significant differences in the effectiveness of different language interventions (additive vs. integrated vs. bilingual/two‐way‐immersion) to promote the L2 development of DLLs?
4. Are language interventions accomplished in ECEC settings more effective in supporting the L2 development (in the society spoken language) of DLLs, when they start early in life (before the age of three)?
5. Are language interventions more effective in supporting the L2 development (in the society spoken language) of DLLs when they are implemented with high fidelity/high quality?
6. Are language interventions with teachers as implementers more effective in supporting the L2 development (in the society spoken language) of DLLs than language interventions with external implementers?
7. Are language interventions with higher intervention dosage (exposure x attention) more effective in supporting the L2 development (in the society spoken language) of DLLs?

## 3 METHODS

### 3.1 Criteria for considering studies for this review

#### 3.1.1 Types of studies

Studies published, unpublished or presented between 1970 and up until 2020 (May 2020) will be eligible for inclusion. Title, Abstract, and/or full text must be available in English or German. So far, no specific systematic review has been completed on the topic. Several systematic efforts of compensatory education and early intervention programs to foster cognitive development of young children at risk started in the early 1970s (Bronfenbrenner, 1974a). In our systematic literature search procedure, we will focus approximately on the last 50 years, beginning with 1970 and the start of the compensatory education in ECEC. The characteristics of educational settings might have changed slightly over time, but policy makers and early education practitioners are still asking the same instructional‐related questions. Is early intervention effective? Which children from what circumstances are most likely to benefit in the short and long run from early interventions? (see Bronfenbrenner, 1974b).

In this review and meta‐analysis, only experimental studies, such as randomized controlled trials (RCT), cluster randomized trials, and quasi‐experimental studies (QED) with equivalent control groups at pretest will be included. Through the information of statistical tests of baseline characteristics, the equivalence of groups will be coded and judged with regards to (a) outcome measures at pretest and (b) child characteristics at pretest (e.g., age, gender, SES). Only studies with equivalent groups of children at pretest will be included.

Wait list control condition, business as usual (regular child care) and alternative treatments (e.g., nonlanguage domain interventions) will be considered as acceptable comparison group. If children in intervention and comparison group are not comparable with regards to baseline measure of outcome, child characteristics or child care setting studies will be excluded. This refers to studies with comparison settings, (a) when children are reared at home and not enrolled in ECEC, because several studies demonstrate a substantial impact of child care attendance on language development of at risk children and DLLs (Anders, 2013; Grob et al., 2014) or (b) when they are enrolled in family child care.

#### 3.1.2 Types of participants

The target population of the review are bi‐ and multilingual children from birth to 6 years of age that are enrolled in ECEC. Consistent with the definition of Child Trends (2014),^1^ DLLs are a diverse group of children. We define DLLs as children who learn multiple (two or more) languages simultaneously and children who make progress on language when they begin to learn a second/further language. Furthermore, children whose parents speak a different language or children who are faced with a different language at home than in society are also considered as DLLs. In particular, DLLs who acquire the second language (society spoken language, L2) in an ECEC setting are at the center of interest. The control group of children must also be DLLs in ECEC. Studies focusing on children with language and speech disorders, or children being at risk for reading disabilities, as well as children with physical, mental, or sensory handicaps are not included in the review.

#### 3.1.3 Types of interventions

In ECEC, several language intervention approaches exist. In the review, intervention studies will be included that measure the impact of a specific language intervention on the minority/family spoken language (mostly L1) and/or society spoken language (mostly L2) development of DLLs within ECEC contexts. The language interventions are required to promote/target the language that is spoken in society (L2). Language interventions can also foster the language that is spoken at home (L1) simultaneously, but L1 promotion is not necessary for inclusion in the review. Language of intervention can be conducted in the society spoken language (L2) or the minority/family spoken language (L1) of the children or trans‐/bilingually. In our definition, language interventions promote oral language skills like expressive and receptive abilities in vocabulary, narration, grammar (syntax and morphology), phonology or pragmatics.

Intervention studies in ECEC that evaluate foreign language learning approaches (that promote development in languages that are not spoken in society, school or family) will be excluded.

Language interventions in ECEC can be categorized in:

1. Additive, pull‐out programs
  Children receive specific instruction/structured intervention. The intervention is created in a pull‐out format, thus activities are applied outside the classroom (separate room) and are not integrated into daily routines. These approaches use small groups or individual lessons. They can either be (A) structured with a manual and material (Sachse et al., 2012) or (B) unstructured/flexible without fix content (Auleear Owadally, 2015).
2. Integrated, interaction‐based strategies in classrooms
  The teacher has a special focus on language supporting interaction strategies (e.g., open questioning, expanding and extending ideas and utterances, corrective feedback, etc.) during daily routines, free play and center time to promote language development in DLLs. This approach can also include (daily) dialogic reading activities (e.g., Schuler et al., 2015). Programs that use only the strategy of enriched teacher–child interactions in book reading sessions and not during other routines and activities would be considered as belonging to (3).
3. Shared book reading
  Children are involved in shared book reading situations which take place regularly (e.g., every day). Teachers use structured interactive techniques to actively engage the children in the text (open questioning, give explanations, draw connections between events in the text and those in the children's own lives as a way of expanding, extending and scaffolding children's utterances). Books can be read with an individual child or with a group of children (Collins, 2010; Ennemoser et al., 2013).
4. Two‐way immersion approaches/bilingual approaches
  Children in the classroom receive language input/exposure in two languages (language of society and the family). At least one teacher in the classroom speaks to children in the family/minority spoken language (L1) or provides instructions in this language during the day or in circle time (Barnett et al., 2007; Buysse et al., 2010).
5. Combination of language support approaches
  This category includes successive or merged combinations of programs as well as combinations of additive and integrated strategies (e.g., RTI or Recognition & Response; LaForett et al., 2013).

#### 3.1.4 Types of outcome measures

The review focuses on the acquisition/development of the societal language (mostly L2) as well as the development of the minority/family spoken language (mostly L1) within the age of 0–6 years. All facets of language outcomes are eligible.

The review will include studies with expressive and receptive measures of vocabulary/semantics, syntax and morphology, phonology, pragmatic abilities, and narrative abilities (e.g, CELF P2, Wiig et al., 2004; CTOPP, Wagner et al., 1998; PPVT, Dunn & Dunn, 2007; WLPB‐R, Woodcock, 1991). Eligible are studies with standardized tests, curriculum based assessments, video ratings, and ratings from parents or teachers. The outcomes may be assessed using standardized or unstandardized instruments. To be included in the meta‐analysis, primary study authors must report enough information to calculate an effect size. If sufficient information to calculate an effect size is not provided, every effort will be made to contact primary study authors and request the necessary information.

##### 3.1.4.1 Primary outcomes

The primary outcomes are the oral language abilities of the societal language (L2), which is primarily acquired within the context of ECEC.

##### 3.1.4.2 Secondary outcomes

The oral language abilities of the minority/family spoken language (L1) of the children can be considered as secondary outcome, since it is not always addresses directly, but could also be profit from general (or sometimes specific) language interventions.

### 3.2 Search methods for identification of studies

We will use four strategies to identify published, unpublished or presented eligible studies:

1. Electronic search
2. Hand search in relevant reviews and reference lists
3. Hand search in relevant journals
4. Hand search in Conference Programs and Proceedings

#### 3.2.1 Electronic searches

We will search in:

- ERIC (via EBSCO)
- Medline (via EBSCO)
- PsycINFO (via EBSCO)
- PSYNDEX (via EBSCO)
- PROQUEST Dissertation and Thesis
- FIS Bildung

For our search strategy we will use, as already described, the PICOC‐System including a clear description of proband, intervention, context, and outcome. We have conducted a pilot search and consulted data‐base‐specific thesauri to locate standardized terms. We will use a combination of terms with truncation (‘ ‘), wildcard (*) and Boolean operators (English: AND, OR, NOT/German: UND, ODER, NICHT). Below are examples of our search terms and combinations.

**Proband:** “dual language learner OR DLL OR ESL OR english as a second language OR bilingual OR EAL OR English as an additional language OR L2 Or Language learner OR immigrant OR English language learner OR limited english speaking OR multilingual OR plurilingual.”

**Intervention:** “language intervention” OR training OR immersion OR promotion OR language foster* OR tuition OR bilingual* OR transition* OR “language learning” OR “language support” OR “language modeling“ OR “language use” OR “second language” OR “language education” OR “language teaching.”

**Context:** preschool OR kindergarten OR prekindergarten OR “early childhood education” OR “early education” OR “early intervention” OR nurser* OR “day care” OR “child care” OR “reception class.”

**Outcome:** language development OR language acquisition OR “second language” OR verbal OR oral OR linguistic OR speech OR language production OR vocabulary OR repetition OR receptive OR grammar OR speak* OR communicat* OR interact* OR proficien* OR utterance.

**Specification:** experimental OR evaluation OR quasi OR intervention study OR between‐design OR “program evaluation.”

**NOT** delay OR impair* OR therapy OR writing OR reading OR “parent training” OR “parent program*” OR deaf OR “down syndrome.”

The combination for the English language Search: (Proband) AND (Intervention) AND (Context) AND (Outcome).

German databases are using completely different thesauri and are restricted to a certain amount of combinations. We will use the following restricted electronic search strategy:

**Proband:** Zweitsprache ODER Mehrsprachigkeit ODER Migrationshintergrund ODER Kinder ODER Vorschulkind.

**Intervention:** Sprachförderung ODER Sprachpädagogik ODER Intervention ODER Effekt ODER Wirkung ODER Wirksamkeit.

**Context:** Kindergarten ODER Kindertagesstätte ODER Frühpädagogik ODER Vorschule.

**Outcome:** Spracherwerb ODER Sprachentwicklung.

The combination for the German language Search: (Proband) AND (Intervention) AND (Context) AND (Outcome).

#### 3.2.2 Searching other resources

##### 3.2.2.1 Hand search in relevant reviews

We will screen the reference lists of prior reviews and meta‐analyses related to language interventions conducted between 1970 and 2020 for additional relevant studies. We identified nine relevant previous reviews and meta‐analyses that might be useful for our search. Two independent coders will screen the reference lists. Further, we will obtain the abstracts of the studies that will be identified as eligible in the previous reviews and meta‐analyses. We will screen the abstracts and examine the relevance of the primary studies related to our topic. We will track the bibliographies of the previous reviews and meta‐analyses (see Appendix A).

##### 3.2.2.2 Hand search in relevant journals

To receive brand new publications and online first papers, we will do an extensive hand search in topic relevant renowned journals. The hand search of titles will include the following journals:

- Early Childhood Research Quarterly
- Early Education and Development
- Early Child Development and Care
- Early Years
- Frühe Bildung

##### 3.2.2.3 Hand search in Conference Programs and Proceedings

Professional meetings and conferences play an important role in the dissemination of scientific information about newly completed research (Garvey & Griffith, 1979; Rosenthal, 1994). Furthermore, the publication process for scientific papers is slow (Rosenthal, 1994). For this reason, attendance at professional meetings and the search of conference programs and proceedings of renowned meetings are appropriate ways to locate information (at least name, title and abstract) of potentially relevant ongoing studies related to our review. To retrieve brand new research results, we will conduct a hand search in conference programs and proceedings for the years 2019 and 2020. We will obtain the PDFs and apply a keyword search with the terms of the electronic search separately. The following international conferences will be taken into account:

- International Symposium on Bilingualism (ISB)
- Society for Research on Educational Effectiveness (SREE)
- European Association for Learning and Instruction (EARLI)
- American Educational Research Association (AERA)
- American Psychology Association (APA)

### 3.3 Data collection and analysis

We have a pool of five trained interdisciplinary coders/reviewers (Ph.D. Education, Ph.D. Psychology, Ph.D. Neuro‐Cognitive Psychology, B.A./M.A. Speech Therapy). Two independent coders/reviewers will carry out the screening and full text coding. Disagreements between the coders have to be resolved through discussion and consensus. Both coders will review the papers simultaneously and discuss them until a final decision is reached.

#### 3.3.1 Selection of studies

A multiple‐step procedure will be applied to identify eligible studies. First, two reviewers will screen the titles and abstracts of all search hits independently using the selection criteria. Then, those full texts that have been judged eligible will be obtained. These studies are called “first‐stage‐eligible studies.” Second, two independent reviewers will code the full texts. Relevance, study quality and eligibility of studies will be judged with our coding schema (see Appendix B). Studies that meet all criteria will be estimated as “relevant” and are called “second‐stage‐eligible studies.” Interrater‐reliability of codings will be estimated with ICC and Kappa to check the quality of codes.

The coding of study will be performed by means of a coding schema (see Appendix B). The first coding schema will be to judge the relevance, study quality and eligibility of relevant studies. The coding categories used here are, besides information regarding bibliographic information and source descriptors (e.g., title, author, publication year, report type, country), study design (e.g., which kind of group comparison, experimental design, mechanism of assignment), sample description (e.g., age, size, selection of participants for intervention), characteristics of intervention and setting (e.g., type of intervention, duration, total length, language, additional components, target group of intervention, age level, implementer), reported outcome measures, and statistical data. Additionally, the bias index will be coded. Here information regarding type of allocation (sample randomization), equivalence of group indicated by statistical data of baseline, drop‐out rate, fidelity of intervention, confounding factors, role of evaluator, reliability of outcome measures, qualification of implementer, blind testing, and effect size will be gathered. The second coding schema (see Appendix B) will be used for extraction of statistical data of “second‐stage‐eligible studies” in order to identify and calculate effect sizes correctly (see next section).

#### 3.3.2 Data extraction and management

Statistical data for effect size calculation and coding at effect size level (see Appendix C) will be extracted from “second‐stage‐eligible studies” only. At effect size level, type of language outcome (L1 or L2), language format (e.g., expressive, receptive) and domain (e.g., vocabulary, morphology), level (e.g., subscale, total score) and type (e.g., standardized test, self‐developed assessment) of measures, align to treatment testing, reliability of used tests, and outcome measures as well as pre‐test differences will be coded.

Two independent coders will apply the extraction of statistical data and coding at effect size level. Disagreements will be resolved through discussion. Interrater‐reliability will be calculated in percentages. Final codes for effect sizes calculation will be entered into CMA.

#### 3.3.3 Assessment of risk of bias in included studies

We will assess the potential impact of *publication bias* using a funnel plot. It is a visual tool in which intervention effect is plotted against its standard error (as an index of precision). Asymmetry may indicate publication bias, where less precise studies (which are smaller studies) are more widely scattered and are assembled at the bottom of the plot. Analogue to the funnel plot, we will apply Egger's regression to estimate a statistical asymmetry (Egger et al., 1997; Sterne et al., 2008). Furthermore, we will carry out a classical fail‐safe *N* analysis to calculate how many studies with null results are needed to reduce the average effect to a nonsignificant value. In addition, a subgroup analysis will be accomplished to compare the effect size of interventions from published and nonpublished reports.

The *influence of method quality* on observed effect sizes are well known. In particular, several bias factors are empirically proofed. Therefore, this meta‐analysis includes a bias index to assess study quality and methodological artifacts. This bias index was developed with regards to the APA Journal Reporting Standards (JARS) for experimental studies (American, 2009), What Works Clearinghouse (2013) Standards and the standards from the German Institute 'Institut für Qualität und Wirtschaftlichkeit im Gesundheitswesen' (IQWiG, 2013). The bias index, with coding at study and treatment level, includes the following criteria:

- Sample randomization,
- Attrition rate (drop out),
- External evaluation to avoid author‐allegiance,
- Reliability of outcome measures, and
- Effect size information/statistical data to calculate effect sizes.

Levels of evidence can be categorized by randomization, attrition, and equivalence (WWC, 2011). In a *randomization procedure study,* participants are randomly (by chance) assigned at students, teachers, classrooms and/or child care centers level either to intervention or control conditions. The unit of analysis “should match the number of “units” that were randomized“ (Deeks et al., 2008, p. 260). More precisely, units of analysis can be biased (a) when groups of individuals (e.g., classrooms, centers) are randomized in the same condition (i.e., cluster‐randomized trials), (b) participants receive a sequence of interventions (i.e., cross‐over trials, multiple treatments), or (c) repeated measures are made. In quasi‐experimental designs, study participants are not randomly assigned and therefore QEDs must demonstrate the *equivalence of intervention and control groups*. High‐quality RCTs also provide information on the equivalence of groups. Further, *attrition rate* (drop out) is an important indicator for levels of evidence and the quality of studies. High attrition rates compromise the initial equivalence of groups and bias effect sizes (cf. What Works Clearinghouse, 2011).

##### 3.3.3.1 Effect size estimation and effect modifiers

Recommendations on the level of evidence for reviewing studies in order to estimate effects more accurately are made by the WWC (What Works Clearinghouse, 2011, 2013). These methodological artifacts will be coded at the effect size level. First, in QEDs, the effect size estimation needs to account for differences in *pre‐intervention characteristics*. Second, for the *clear attribution of the effect on the intervention*, the intervention cannot be combined with other interventions. Third, the intervention must be *implemented as designed* (indicating high fidelity) to measure an effect. Forth, the *unit of analysis* must be considered when examining the impact (cf. What Works Clearinghouse, 2011). These recommendations provide methodological issues that will be tested as moderators in the forthcoming meta‐analysis.

At effect size level, a methodologically based bias may occur with regards to:

- Measurement features,
- Outcome construct,
- Source of information, and
- Origin of measures.

##### 3.3.3.2 Measurement features

Measures must be valid, reliable, and age‐appropriate (What Works Clearinghouse, 2011; Yoon et al., 2007). The *psychometric properties* of outcome measures must be adequate to capture the impact of professional development on quality and student gains, and information on reliability must be provided (cf. What Works Clearinghouse, 2011). In particular, effect size calculations were found to vary significantly according to the reliability of the scales used (cf. Beelmann, 2014; Hunter & Schmidt, 2004).

Further, *outcome construct* was associated with variance in effect sizes (Wilson & Lipsey, 2001). Even within an outcome domain (e.g., language), the treatment effects were typically much larger for some outcome variables than for others (e.g., vocabulary vs. grammar). It is not surprising that treatments have a larger impact on some variables. However, Wilson and Lipsey (2001) show that different outcome constructs within one domain explained seven percent of the variance (p. 418).

The *source of information* (e.g., teacher or parent ratings, observations, test scores) may confound the effect size calculations. Five percent of the variance in effect size estimates were explained when measures were grouped for source of information, including ratings, observations and physical measures (Wilson & Lipsey, 2001). The study by Harvey et al. (2013) investigated predictors of discrepancies between mothers', fathers', and teachers' ratings on behavioral aspects (e.g., hyperactivity, attention problems, aggression) among 3‐year olds. Mothers', fathers', and teachers' ratings differed significantly and were predicted for example by ethnicity, ADHD/ODD diagnoses, parental depression, number of children and pre‐academic skills.

The *origin of measure*, which means whether the instrument/scale was developed by the researcher or preexisted as a standardized/published instrument, was tested as a potential effect modifier. Variability due to the origin of the measures was found predominantly in educational research, where researcher‐developed measures yielded larger effect sizes (Wilson & Lipsey, 2001). It has been suggested that instruments should be sensitive to identify changes and measures should be aligned to interventions to detect effects (Yoon et al., 2007). Researcher‐developed measures might capture more relevant aspects of changes due to interventions, but are hardly comparable and might lack appropriate reliability.

#### 3.3.4 Measures of treatment effect

##### 3.3.4.1 Treatment effects must be measured trough language development in L1 or L2

*L1 development* of DLLs can be measured in different ways. Standardized tests and assessments are available only for a limited amount of languages (e.g., Spanish, Russian, Turkish, French). Tests can be administrated by native‐speaking evaluators (e.g., PPVT, Dunn & Dunn, 2007; TVIP, Dunn et al., 1986). Alternatively, computer assisted assessments for some languages exists (e.g., SCREEMIK 2 for Turkish and Russian children, Wagner, 2008). Further, parental ratings on L1 development are eligible, because of the lack of standardized instruments.

*L2 acquisition and development* of DLLs should be measured through objective procedures and reliable instruments. Therefore, standardized tests accomplished by assessors or parents are eligible. Quantity and quality of utterance of children (e.g., syntax complexity, MLU) can be captured and investigated through video analyses.

In sum, studies must report at least one of the following outcomes of expressive or receptive abilities in:

- Vocabulary,
- Narrative skills,
- Grammar (syntax and morphology),
- Phonology, and
- Pragmatics.

#### 3.3.5 Unit of analysis issues

We will use an independent sample as the unit of analysis (Cooper, 2010) to integrate effect sizes. If one report contains two or more separate studies based on different samples, we will count each separate study (associated with each respective independent sample) as a unit. Further, if one study contains two or more separate treatments and an additional control group, we will count each treatment separately. To ensure that the control group is not over‐weighted in the meta‐analysis, we will divide the number of participants in the control group according to the number of treatments in the study. As a result, the number of independent samples/treatment‐control‐group comparisons might be greater than the number of eligible studies reported in the review/meta‐analysis. We will utilize multilevel meta‐analyses to help handle effect size dependency issues arising from the same study.

In such cases where *several studies are described in one single report*, each study will be coded separately and will receive a separate study number.

Within one study, it is likely that *several control groups* are available. Only the intervention and control groups that meet the eligibility criteria will be included. If several control or comparison groups are present, we will choose the most untreated condition. If only marginally treated control groups (e.g., teachers receive a workshop on language acquisition, teachers receive books, teachers are told to spend extra time during daily routines with target control children) are available, we will code them as alternative treatment control conditions. Control conditions (e.g., waitlist, placebo) will be coded separately and different control conditions will be tested in the moderator analysis.

Some of the studies compare *multiple experimental groups* with the same control group. For coding purpose, we will make separate coding sheets for each treatment and control comparison. Different treatments receive separate treatment numbers that are nested under the study and finding number. However, correlations between the multiple comparisons and their outcomes are rarely reported. Thus, we will include these studies in the analyses assuming a zero correlation between the outcomes. To avoid the violation of the assumption of independency, the sample of the control group will be divided for effect size calculation (for detailed information see effect size calculation).

When a study has *multiple indicators* for the same construct (for instance more than one global measure of language development), the effect size of all indicators will be coded and used to calculate effect sizes. Effect sizes will be coded by instrument, outcome domain (e.g., receptive vocabulary, expressive vocabulary) and level of measure (e.g., total score, subscale, item). For every effect size, we will code the specific outcome domain (e.g., expressive vocabulary in L2) and create separate data sets for each language. As a consequence, data sets for each language domain will contain different numbers of studies. If enough studies are available, we will conduct multilevel aggregation of effect sizes at a composite level (e.g., language comprehension in L2).

When a study has *multiple time‐points*, we will use pretest and posttest measures that must be conducted during ECEC (before first grade) to calculate intervention effects in the early years. We will separate short term interventions that are conducted over a couple of weeks in comparison to comprehensive programs that run during the whole school year. Follow up measures can also be accomplished with school aged children in elementary schools. We will also use follow up measures to estimate sustainability effects and to analyze if there are fade or sleeper effects. Here the amount of time (in months) between posttest and follow up will be coded as potential effect modifier.

#### 3.3.6 Dealing with missing data

Full text of relevant studies will be obtained from the library system. Further, unpublished, in‐press reports or conference slides will be requested by contacting the corresponding authors. In addition, we will contact leading authors through e‐mail and Research gate for missing information (e.g., statistical data). In cases in which more than one report describes study findings that are all based on the same sample, we will choose the study report of the sample with the best design and/or psychometric quality and/or most information on treatment and moderators. If information is missing on theoretical and methodological effect moderators, we will extract these data from the other study related reports and include them in the coding schema.

#### 3.3.7 Assessment of heterogeneity

The *Q*‐statistic will be used as the *test of heterogeneity*. *Q* is sensitive to the ratio of observed variation in within‐study error. “The *Q* test is computed by summing the squared deviations of each study's effect estimate from the summary effect estimate, weighting the contribution of each study by its inverse variance" (Huedo‐Medina et al., 2006, p. 4). When all studies share a common effect size (null hypothesis), *Q* follows a *χ* ^2^ distribution with the degree of freedom of *k* − 1, where *k* is the number of studies (Deeks et al., 2008). A significant *p* value (rejecting the null hypothesis) provides evidence for some variance in the true effect sizes and supports the rationale for applying a random‐effect model (Huedo‐Medina et al., 2006). The *I* ^2^ statistic represents the proportion of observed variance that reflects real differences in effect sizes (formula: *I* ^2^ = ((*Q* ^2^ − *df*)/*Q*) × 100). It reflects the extent of overlap of confidence intervals and quantifies the inconsistencies (Deeks et al., 2008). According to Deeks et al. (2008, p. 278), the *I* ^2^‐statistic can be interpreted as follows:

0%–40% might not be important;
30%–60% represent moderate heterogeneity;
50%–90% represent substantial heterogeneity;
75%–100% represent considerable heterogeneity.

Further, between‐study variance in effect sizes will be estimated with Kendall's^2^ (Borenstein et al., 2009).

#### 3.3.8 Assessment of reporting biases

A meta‐analysis is a mathematically accurate synthesis of a set of studies. If the studies are based on a biased selection of investigations then the computed effect will reflect this bias. The issue that published studies are more likely to be integrated in meta‐analyses is called publication bias or the file drawer effect (cf. Borenstein et al., 2009; Rosenthal, 1979). In addition, small studies without significant effects are more likely to remain unpublished. Further, the tendency for effect size estimates in small studies to differ from those in large studies is called the small‐study effect. In particular, poor methodological quality leads to inflated effects in smaller studies (Sterne et al., 2008). The precision of effect size estimates increases as the size of studies increases (Harbord et al., 2009). Funnel plots can be used to display the relationship between sample size or standard error (*y* axis) and effect size (*x* axis). A file drawer problem is assumed when there is asymmetry with many small studies lying at the bottom of the graph and spread around the full range of the graph (Borenstein et al., 2009; Harbord et al., 2009). The interpretation of plots seems to be largely subjective. To investigate the file drawer problem, the Egger regression uses a linear regression approach to measure funnel plot asymmetry (Borenstein et al., 2009; Egger et al., 1997). It is proposed that researchers “perform a linear regression of the intervention effect estimates on their standard error, weighted by 1/(variance of the intervention effect estimates)” (Sterne et al., 2008, p. 314). A straight‐line relation between intervention effects and standard errors is assumed and the intercept provides a measure of asymmetry, where a larger deviation than zero indicates a more pronounced asymmetry (Egger et al., 1997).

#### 3.3.9 Data synthesis

For *effect size aggregation*, we will use a random effect model. For each language domain (e.g., receptive vocabulary or expressive vocabulary) a separate data set will be conducted. To estimate a precise summary effect size with minimum variance, weighted means will be calculated, where “the weight assigned to each study is the inverse of the study's variance” (Borenstein et al., 2009, p. 72). Within a random effect model, it is assumed that the effect in different studies is similar, but not identical, and follows the same distribution. In particular, this occurs when studies differ (1) in the mixture of participants (e.g., language groups), (2) in intervention implementation, or (3) in the use of different measures (Borenstein et al., 2009). Assessing the impact of educational interventions, the magnitude of effects might vary depending on characteristics of the educational settings such as class size, age of children, or proportions of disadvantaged children in the classroom. Educational setting factors are likely to vary from study to study. Therefore, random effect models will be used. The theoretical reason behind this is that different population distributions can be assumed due to the fact that the studies included are based on various outcome measures and multiple in‐service training approaches in various ECEC settings.

When enough studies are available, we will use a multilevel approach to aggregate the findings at composite levels for language category (e.g., language comprehension vs. language production). Multilevel analysis acknowledges the hierarchical structure of effect sizes nested under studies (Raudenbush & Bryk, 2002). For example, in measuring the impact of language interventions on L1 and L2 abilities, various specific domains of language are assessed within studies (e.g., vocabulary, syntax, morphology). To aggregate such different outcome measures into a summary effect size, a multilevel approach is suggested (Hedges, 2009; Raudenbush & Bryk, 2002). Specifically, a 2‐level approach will be used to consider variations in effect size and treatment level.

#### 3.3.10 Subgroup analysis and investigation of heterogeneity

The variation in outcomes across studies can be analyzed through meta‐regressions and subgroup analyses, which test for potential moderators (Raudenbush & Bryk, 2002). We will use a two‐step procedure: (1) First, single bivariate analyses will be accomplished for all reliable coded moderators. However, this procedure of multiple‐testing of different subgroups and linear relations might imply an alpha‐error‐inflation. (2) Second, multivariate regressions will be calculated including significant theoretical and empirical moderators to avoid the alpha‐error‐cumulation. However, the use of multivariate analyses is entirely dependent on the number of studies that are appropriate for the meta‐analysis and the coherent information on specific moderators. Nevertheless, multivariate models are the best choice to estimate unbiased effect sizes and draw evidence‐based conclusions for research and practice.

*Subgroup analyses* will be used to investigate differences for subgroups and/or for categorical or dummy coded variables. Then the regression coefficient shows how the intervention effect differs between reference groups and the significance test indicates statistically considerable differences (Deeks et al., 2008, p. 285). *Q* and *p* values are two key statistics in the subgroup‐analysis. The *Q* represents the total between‐group variance associated with the subcategories of the moderator variable. The *p* value is the result of a significance test for the mean difference between or among the subgroups.

In general, we will avoid conducting moderator analyses on too many variables, in particular when the number of eligible studies is limited. We will apply subgroup analyses for methodological moderators (e.g., randomization, external evaluation, source of information, or origin of measure) and intervention moderators with regards to type of intervention, fidelity, implementer, and start of intervention (research question 3, 4, 5, and 6).

*Meta‐regression* allows researchers to investigate the effect of a single moderator or multiple factors simultaneously. It explores the relationship between continuous variables (e.g., age, intervention dosage) and the mean effect size. The outcome variable (effect size) can be predicted by one or more explanatory variables. In particular, potential effect modifiers (characteristics of studies) that predict treatment effects can be examined, when there are more than ten studies included (Deeks et al., 2008). The obtained regression coefficient describes how the intervention effect changes with a unit increase in the explanatory variable (effect modifier) and the statistical significance shows the linear relation between effect and modifier. When enough studies and data are available, we can use meta‐regressions to analyze several moderators simultaneously. We will apply meta‐regression for methodological issues (e.g., number of participants, age of sample) and for the analysis of intervention dosage and intensity (research question 7).

#### 3.3.11 Sensitivity analysis

Sensitivity analysis will be conducted to estimate the stability of the average effect size. In particular, a one‐study‐remove analysis will be used to estimate the stability of the average effect size, where one treatment (as unit of analysis) is removed to identify outlier studies. In addition, Classical and Orwin Fail Safe *N* analyses will be carried out to calculate how many treatments with null results (mean effect of zero) are needed to bring the average effect down to a nonsignificant value of *g* < 0.20 or 0.

#### 3.3.12 Summary of findings and assessment of the certainty of evidence

##### 3.3.12.1 Summary of findings

First, the findings will be aggregated in two separate meta‐analyses. The effectiveness of language promotion in ECEC will be summarized in findings on (1) the societal language (L2) and (2) on the minority/family spoken language (L1) of children.

**Table 2:** Example for the structure of the “summary of findings” table

Outcomes	Effect (95% CI)						# of participants (Studies)	Certainty of evidence	Comments
Vocabulary	Embedded strategies	Specific curriculum	Small groups	Shared book reading	Bilingual programs	Combination of approaches	◯◯◯◯		

##### 3.3.12.2 Assessing the certainty of evidence

The certainty of evidence will be statistically assessed for each outcome and subcategory with regards to the GRADE dimensions (Hultcrantz et al., 2017). In particular, (1) the risk of bias (e.g., selection bias, researcher allegiance, small study effects), (2) publication bias, (3) the precision of effect estimates, and (4) the consistency of individual study results will be analyzed through meta‐analytic techniques (e.g., *Q*‐ and *I* ^2^‐statistics, *p* values, 95% confidence interval, meta‐regression, subgroup analysis, egger regression).

Further, we will judge (5) the directness of evidence (whether the available data is directly related to answer the question of interest) coding and analyzing different aspects of:

(a) study population (e.g., SES, age, language group),
(b) intervention (e.g., region, child care setting)
(c) outcome (e.g.,. productive and receptive skills, assessment procedure and reliability)
(d) comparison condition (e.g., type of comparison).

However, the aspects that are used to judge directness of evidence are completely dependent on the information that is available in the primary research papers.

## CONTRIBUTIONS OF AUTHORS

Franziska Egert is responsible for the data management, the extracting of data from papers, and the analysis of data. Steffi Sachse is responsible for appraising the quality of the retrieved papers and providing general advice on the review. Katarina Groth is also responsible for appraising the quality of the retrieved papers and for organizing the retrieval of papers and the screening of search results. The authors contribute equally to the project by designing and coordinating the project, writing the protocol and review, coding of studies, and interpreting the results.

## DECLARATIONS OF INTEREST

All authors published research papers in the area of review. The authors are all experienced researchers in the field of language and literacy development and externally evaluated the impact of different language interventions (e.g., KIKUS or Vorlaufkurs Deutsch) and governmental strategies. Further, two authors developed in‐service teacher trainings in the area of language development (e.g., HIT or VERBAL*).

All authors assure that there is no potential conflict of interest. The systematic review and the meta‐analysis will be conducted with regards to the standards and guidelines of the Campbell Collaboration and the Cochrane Collaboration with high quality, rigor and research objectivity.

## SOURCES OF SUPPORT

### Internal sources

- State Institute of Early Childhood Research, Germany.
  Providing the infrastructure to realize the project.

### External sources

- Thomas Mangold, Director of the University Library, Catholic University of Applied Science Muenchen, Campus Benediktbeuern, Germany.
  Providing consultation with regards to electronic search procedure.

## Footnotes

^1^
There is no single consistently used definition of DLLs. The one used here takes an inclusive approach, counting as DLLs all children living in a household where one or more members speak a language other than English. We assume that these children, in addition to their exposure to a non‐English language, have some degree of exposure to English” (Child Trends, 2014. Databank indicators. Dual Language Learners. Available at: http://www.childtrends.org/?indicators=dual-language-learners).

## APPENDIX A LIST OF PREVIOUS REVIEWS

Bengochea, A. (2014). *The effects of vocabulary instruction on bilingual students' lexico‐semantic acquisition in English‐Medium contexts: A systematic analysis*. University of Miami.

Buysse, V., Peisner‐Feinberg, E., Páez, M., Scheffner Hammer, C., & Knowles, M. (2014). Effects of early education programs and practices on the development and learning of dual language learners: A review of the literature. *Early Childhood Research Quarterly*, 29(4), 765–785.

Chambers, B., Cheung, A., Slavin, R. E., Smith, D., & Laurenzano, M. (2010). *Effective early childhood education programs: A systematic review*. Center for Research and Reform in Education. http://www.bestevidence.org/word/early_child_ed_Apr_15_2010.pdf

Duggan, B., Dosmukhambetova, D., & Edwars, V. (2014). *A review of research evidence on the effectiveness of different approaches to promoting early speech and language development*. Cardiff: Welsh Government Social Research. http://dera.ioe.ac.uk/20176/1/140528-evidence-effectiveness-different-approaches-promoting-early-speech-language-development-en.pdf

Durán, L. K., Hartzheim, D., Lund, E. M., Simonsmeier, V., & Kohlmeier, T. L. (2016). Bilingual and home language interventions with dual language learners: A research synthesis. Language, *Speech, and Hearing Services in Schools*, 47(4), 1–25.

Marulis, L. M., & Neumann, S. B. (2010). The effects of vocabulary intervention on young children's word learning: A meta‐analysis. *Review of Educational Research*, 80, 300–335.

Mol, S. E., Bus, A. G., & De Jong, M. T. (2009). Interactive book reading in early education: A tool to stimulate print knowledge as well as oral language. *Review of Educational Research*, 79, 979–1007.

What Works Clearinghouse. (2007). *Dialogic reading*. Washington D.C.: Institution for Education and Sciences. https://ies.ed.gov/ncee/wwc/Docs/InterventionReports/WWC_Dialogic_Reading_020807.pdf

What Works Clearinghouse. (2010). *Dialogic reading*. Washington D.C.: Institution for Education and Sciences. https://ies.ed.gov/ncee/wwc/Docs/InterventionReports/wwc_dialogic_reading_042710.pdf

What Works Clearinghouse. (2015). *Shared book reading*. Washington D.C.: Institution for Education and Sciences. http://ies.ed.gov/ncee/wwc/pdf/intervention_reports/wwc_sharedbook_041415.pdf

## APPENDIX B CODING MANUAL (SHORT FORM)

1. **Date** [date]: _______________
2. **Coder ID** [coder_id]: _______________
3. **Study ID number** [studyid]: _______________
4. **Finding Number** [findnr]: _______________
5. **Treatment Number** [treat_nr]: _______________
6. **Author** [auth]:____________________________________________________
7. **Title of Publication** [title]: ______________________________________________________________________
8. **Publication year** [pubyr]: _______________
9. **Country study was accomplished in** [country]:____________________________
10. **Type of publication** [publish]

(1) published (e.g., article, book, chapter)

(2) unpublished (e.g., report, dissertation)

**11. Type of study** [st_type]: page(s): _____

(1) program with control/comparison group (child care as usual)
(2) program without control/comparison group
(3) comparison of two treatments (alternative language intervention e.g., unstructured small group intervention or short‐term teacher workshop)
(4) comparison with placebo treatments (placebo = non‐language intervention)

**12. Study design** [st_design]: page(s): _____

(1) experimental
(2) quasi‐experimental
(3) correlational (results only providing correlational data)
(4) one group longitudinal (non‐experimental)
(5) single subject design/multiple baseline

**13. Mechanism of allocation** [assign]: page(s): _____

(1) random assignment at individual level
(2) random assignment at group/center level
(3) non‐random assignment, but control group matched to treatment group
(4) non‐random, no matching prior to treatment (groups of convenience)
(5) assignment to condition not described or vague
(6) study without experimental design/no assignment to conditions

**14. Sample size at beginning (pre‐test data)** page(s): _____

IG 1: ________ [exgr1] CG: _______ [congr1] total sample: _______ [tosamp]

**15. Age of children** [sampleage] page(s): _____

**Table:**

	Intervention group	Control group	Total sample
M age (in Months)			
SD			

Age range: ________________months

◯ long term effects (data with follow up at school age?)

◯ only long term effects (data for T2 in school age/no T2 data in preschool/kindergarten)

**16. Name of intervention** [nameintervention]:

_____________________________________________________________________________

**17. Target group of intevention** [target]: page(s):_____

(1) monolingual children
(2) all children in center based care (monolinguals and multilinguals)
(3) all children with language difficulties
(4) dual language learners
(5) dual language learners with language difficulties
(6) no information

**18. Selection of intervention children** [select_intervention] page(s):_____

(1) through standardized assessment
(2) through teacher suggestion by checklist/observation (e.g. Sismik)
(3) through teacher subjective suggestion
(4) no selection mechanism/groups of convenience
(5) randomization procedure

**19. Sample description** [sample_type]: page(s):_____

(1) monolingual children
(2) all children in center based care (monolinguals and multilinguals)
(3) all children with language difficulties
(4) dual language learners
(5) dual language learners with language difficulties
(6) no information

**20. Program type** [program]: page(s): _____

(1) pull out program without manual (e.g. naturalistic small group intervention)
(2) (structured) pull out training program with manual & material
(3) integrated & embedded language intervention (improved language stimulation in routines)
(4) shared book reading/dialogic reading
(5) immersion program
(6) digital teaching (apps, audio books, computer programs, television)
(7) combination: ____________________________________________________
(8) bilingual dual/transitional program
(9) no detailed information available

**21. Duration of intervention** [li_duration] page(s): _____

_____ total duration in months (end – beginning); Calculation: _______________(school year=9 months)

**22. Total length of intervention** [li_length] page(s): _____

_____ total length of training in hours; Calculation: ____________________________

**23. Age group for intervention at beginning** [targetage]: page(s): _____

(1) children under 3
(2) children above 3
(3) year before schooling (e.g. kindergarten, 5‐6 years old)
(4) mixed age intervention (e.g. 2‐4):_________________years

**24. Additional component of language intervention** [supplements] {Code 1=yes, 0=no} page(s): _____

◯ parental involvement? How?_________________________________________________________

◯ fostering of L1 development? How?____________________________________________________

**25. Target outcome measures of intervention** [target_outcome] {Code 1=yes, 0=no} page(s): _____

◯ L1 development measures

◯ L2 development measures

◯ literacy measures (letter & alphabet, early reading & writing, phonological awareness)

**26. Language of intervention** [languageofintervention] {Code 1=yes, 0=no} page(s): _____

◯ L1:minority/family spoken language

◯ L2:majority/society spoken language

◯ L2:foreign language

**27. Implementer** [implementer] {Code 1=yes, 0=no} page(s): _____

◯ teacher

◯ external implementer

**28. Outcome measures** [outcomen]: {Code 1=yes, 0=no} page(s): _____

◯ standardized test/assessment

◯ curriculum aligned test

◯ external observation

◯ teacher rating

◯ parental rating

Instruments: _________________________________________________________________________

**29. Statistical (M, SD, N or ES,N) information available?** page(s): _____

(1) yes, data available to estimate effect sizes

(0) no, data available to estimate effect sizes

**30. Relevance for meta‐analysis** [relevance]:

(1) relevant

(0) not relevant

*Notes*:

**BIAS INDEX**

**1**. **Randomization page**(s): ___

Children were randomized allocated to treatment or control group (individual or classroom level).

(1) yes

(0) no

(9) no information

**2. Equivalence of groups** {Code 1 = yes, 0 = no} page(s): ____________________________________

(1) yes, IG and CG are equivalent on structural features of child (e.g., gender, age, language)

(2) yes, IG and CG are equivalent on structural features of family (e.g., SES, education)

(3) yes, IG and CG are equivalent on structural features child care (e.g., ratio, teacher education)

(9) no information available

**3. Drop out** page(s): _______________________________________________________________________________% Drop out (pre to posttest)    _____________% Drop out (posttest to follow up)    ____________________matched group

**4. Fidelity** page(s): __________________________________________________________________

Fidelity of interventions was measured as high? (1) yes     (0) no     (9) no information

How was fidelity measured?___________________________________________________________

How was high fidelity guaranteed? (e.g., certified, experienced trainers, extra training, ongoing support) __________________________________________________________________________

**5. Confounding factors** page(s): ________________________________________________________

(0) no

(1) yes, confounding factors: _____________________________________ (e.g., treatment/material)

**6. Extern evaluation** page(s): __________________________________________________________

(1) yes, intervention was investigated by external evaluators

(2) no, implementers or authors of the program are evaluators

(9) no information available

**7. Reliability of outcome measures** {Code 1 = yes, 0 = no} page(s):____________________________

(1) yes, standardized test

(2) yes, good reliability scores (α > .65)

(3) yes, good inter‐rater‐reliability scores (Kappa oder ICC > .70)

(4) no, bad reliability scores/no information

**8. Blind testing** (child development) page(s): _____________________________________________

(1) yes, assessors/tester were reported as blind

(9) no information

**9. Effect sizes** page(s): _______________________________________________________________

(1) yes, effect size is reported (Cohen's d or Hedges'g)

(9) no information

**10. Setting** {Code 1 = yes, 0 = no} page(s): ________________________________________________

(1) Setting with children at risk (e.g. Head Start, High Scope)

(2) Setting with children under the age of 3 (infant/toddler)

**11. Percentage of intervention implementers with academic degree** (BA or higher) page(s):_______________% in total sample   _______ % in intervention group   _________ % in control group

## APPENDIX C EFFECT SIZE CODING

Effect sizes are calculate with the Comprehensive Meta‐Analysis (CMA) software (Borenstein et al., 2005). CMA calculates effect sizes from more than 100 types of data formats on the basis of a wide variety of statistics. At effect size level, we also code the following items:

### 1. Type of language outcome

(1) L1: family spoken language
(2) L2: society spoken language

### 2. Language format

(1) expressive/productive skills
(2) receptive skills
(3) mixed composite score/total score “language”

### 3. Language domain

(1) vocabulary
(2) narrative skills
(3) grammar (syntax and morphology)
(4) phonology
(5) pragmatics

### 4. Level of measures

(1) item
(2) subscale
(3) total score/composite score

### 5. Type of measure

(1) standardized test
(2) curriculum based assessment
(3) self‐developed assessment
(4) video rating
(5) teacher rating
(6) parental rating

### 6. Test‐Alignment to treatment

(0) no, standardized

(1) yes, curriculum based/test or items aligned to treatment

### 7. Reliability of outcome measures

(0) no information/no standardized test/not reliable (α < .65)

(1) no specific information on this sample, but standardized test was used

(2) yes, outcome measure is described as reliable (α < .65) with the study sample

### 8. Pretest differences

(0) equal scores of intervention and control group

(1) control group had significant higher scores at pretest

(2) intervention group had significant higher scores at pretest
